# Vector-borne pathogens affecting shelter dogs in eastern Crete, Greece

**DOI:** 10.1007/s00436-019-06284-z

**Published:** 2019-03-13

**Authors:** Mathias Hofmann, Adnan Hodžić, Niki Pouliou, Anja Joachim

**Affiliations:** 1 Institute of Parasitology, Department of Pathobiology, University of Veterinary Medicine Vienna, Veterinaerplatz 1, 1210 Vienna, Austria; 2 Animal Medical Center, Kýprou 61, 722 00 Ierapetra, Greece

**Keywords:** Arthropod-borne, Blood-borne, Shelter, *Leishmania*, *Hepatozoon*, *Mycoplasma*

## Abstract

Canine pathogens transmitted by blood-sucking arthropods are of significant importance for veterinary and, in some cases, human health. However, they are still underestimated and rarely investigated in many parts of the Mediterranean region, mostly due to financial reasons. Therefore, in the present paper, we investigated the occurrence of blood-associated pathogens affecting dogs in Crete, Greece. For this purpose, blood samples from 103 shelter dogs were screened for the pathogens by PCR and serological tests. Overall, samples from 43 dogs scored positive for at least one pathogen (41.8%). In particular, antibodies to *Leishmania infantum* were detected in 26 dogs (25.2%), and 15 and 11 animals were positive for *Hepatozoon canis* (14.6%) and *Mycoplasma haemocanis* (10.7%) by PCR, respectively. Co-infections were recorded in nine animals. Clinical signs indicative of infection (alterations of skin or coat or reduced body condition) were detected in 10 animals, four of which were infected with one pathogen, three with two pathogens. Based on the results obtained, dogs from Crete appear to be frequently exposed to several blood-borne pathogens, including agents of zoonotic concern. Given that some of the pathogens were reported for the first time in this area, results presented in our study should improve the awareness of the local veterinarians and of dog rescue organisations in order to reduce disease burden on stray and owned dogs and to control the spread of canine vector-borne diseases from Greece to non-endemic areas by travelling or exported infected dogs.

## Introduction

Canine vector-borne diseases (CVBD) are caused by a range of infectious agents, transmitted by blood-feeding arthropods, including ticks, mosquitoes, fleas, and phlebotomine sandflies. In addition to their veterinary importance, some of the pathogens are of a major zoonotic concern and may even cause severe and life-threatening diseases in humans (Otranto et al. 2009). The global distribution and unspecific clinical presentations in infected animals make the diagnosis and control of CVBD highly complex and challenging (Miró et al. 2013). Stray and free-roaming dogs are particularly at high risk of infection due to a lifestyle that involves frequent exposure to competent arthropod vectors and poor veterinary health care, and consequently they represent a permanent source of infection for the vectors and indirectly for other animals and humans (Otranto et al. 2009; Diakou et al. 2018). Expansion of CVBD and their vectors could in principle be attributed to climate changes and habitat changes, but increasing pet tourism and export of stray dogs from endemic to non- or low-endemic countries have been confirmed as an efficient route of pathogen dissemination (Menn et al. 2010; Otranto et al. 2009).

Over the past decades, an increasing number of scientific reports of CVBD in both pet and stray dogs in Europe have been published. However, data on their distribution are still not available for all endemic areas, including parts of insular Greece, where CVBD are assumed to be highly prevalent. A more recent study showed that up to 65% of dogs living on four islands of Greece (Santorini, Tinos, Ios, and Skiathos) were infected with vector-transmitted pathogens, and most of them have a zoonotic potential (Diakou et al. 2018). Since data from Crete, the largest Greek island, are missing, we aimed to investigate the occurrence of common blood-borne protozoa (*Leishmania*, *Babesia*, *Hepatozoon*), bacteria (Anaplasmataceae, hemotropic mycoplasmas), and filarioid nematodes in dogs from shelters.

## Material and methods

**Fig. 1:**
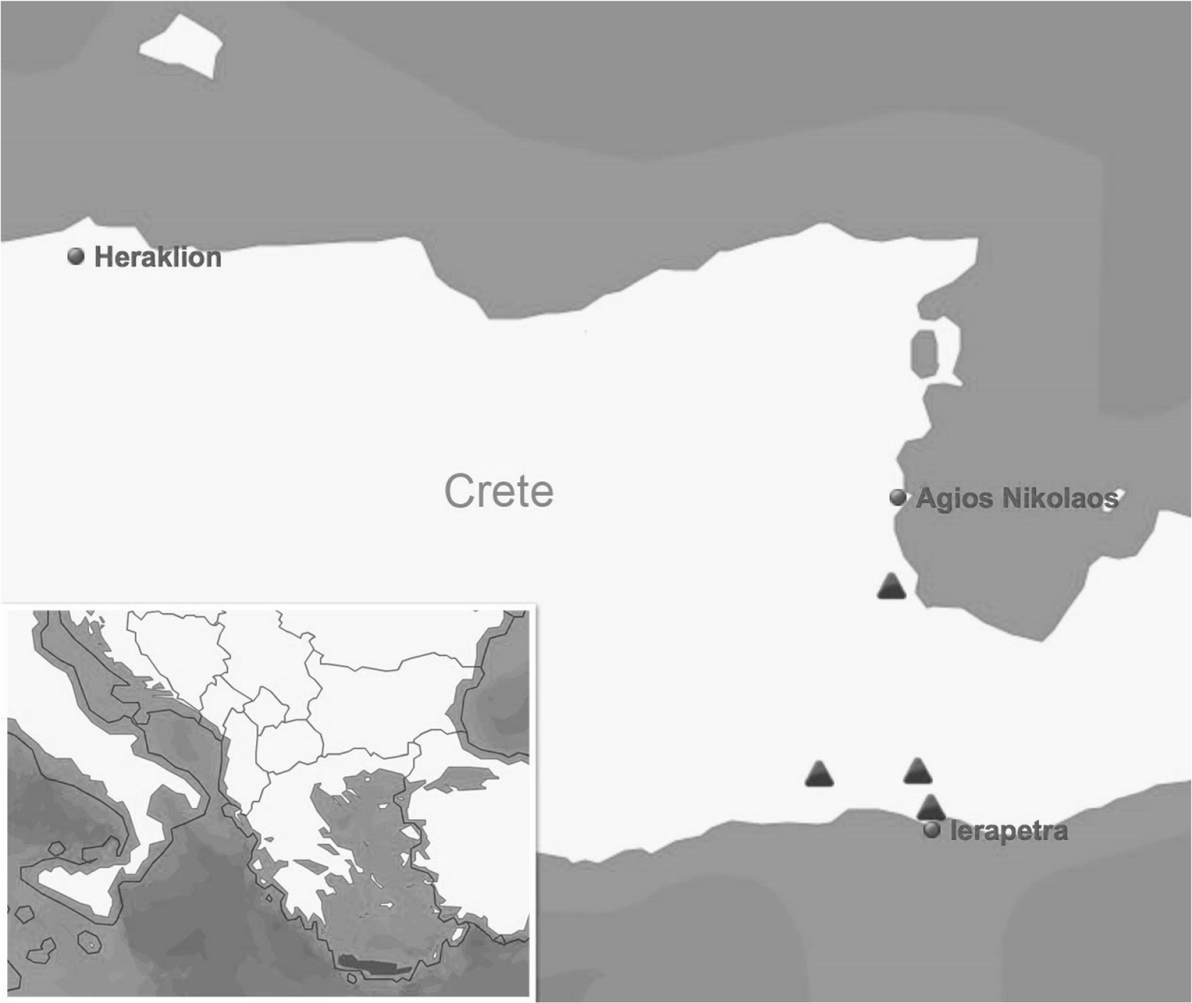
A map of Greece showing locations of the dog shelters involved in the study

In December 2017, four animal shelters (Fig. 1) in eastern Crete were contacted and asked to participate in this prospective study. The majority of animals were ownerless and taken to the shelters from streets, and thus were most likely not vaccinated and not treated against parasites on a regular basis. In total, 103 dogs of different breeds, sex, and age were included in the study. The animals were checked for the presence of ectoparasites and clinically examined for signs indicative of CVBD (e.g., anorexia, lethargy, pale mucous membranes, dermatological and ocular alterations, hyperthermia, or enlarged lymph nodes). Blood samples for molecular and serological analyses were taken from available dogs from the cephalic or lateral saphenous vein. Quick tests (SNAP® *Leishmania* and SNAP® Heartworm RT tests, IDEXX Veterinary Diagnostics, Westbrook, ME) were performed on the sampling day to detect antibodies against *Leishmania infantum* or antigen of the heartworm *Dirofilaria immitis*. Whole blood was stored at − 18 °C until used for molecular analyses. All sampling procedures were routinely performed by veterinarians on study sites, and the work was approved by local authorities. No further ethic permissions were required as no additional samples (besides the ones for routine health inspection of incoming animals) were taken for this study and the supervising veterinarian (NP) participated in the study.

DNA was extracted from 200 μl EDTA-blood with a High Pure PCR Template Preparation Kit (Roche Diagnostics GmbH, Germany) following the manufacturer’s instructions. Targets and primer sequences used for molecular detection of the tested pathogens are listed in Table 1. PCR products were separated using 2% agarose gel stained with Midori Green Advance DNA (Nippon Genetics Europe, Germany). Purification and sequencing of the amplicons were carried out by a commercial provider (LGC Genomics, Germany). Obtained nucleotide sequences were edited with BioEdit software v.7.2.5 (Hall 1999) and then checked for similarity with those available in the GenBank® database (http://www.ncbi.nlm.nih.gov/BLAST).

**Table 1:** Primer sets and protocols used for PCR amplification of blood-borne agents in dogs from Crete, Greece

Target organism	Genetic marker	Primer sequences (5′ → 3′)	Product size (bp)	Reference
*Leishmania infantum*	kDNA	RV1: CTTTTCTGGTCCCGCGGGTAGG RV2: CCACCTGGCCTATTTTACACCA	145	le Fichoux et al. 1999
	ssu-rRNA	Lei70L: CGCAACCTCGGTTCGGTGTG Lei70R: CGCGGTGCTGGACACAGGGTA	345	Spanakos et al. 2002
	*cpb*	Leicpb_for: CGTGACGCCGGTGAAGAAT Leicpb_rev: CGTGCACTCGGCCGTCTT	702	Hide and Bañuls 2006
*Babesia/Hepatozoon*	18S rRNA	BTH-1F: CCTGAGAAACGGCTACCACATCT BTH-1R: TTGCGACCATACTCCCCCCA Nested GF2: GTCTTGTAATTGGAATGATGG GR2: CCAAAGACTTTGATTTCTCTC	720/750 590/610	Zintl et al. 2011
*Hepatozoon* spp.	18S rRNA	H14Hepa18SFw: GAAATAACAATACAAGGCAGTAAAATGCT H14Hepa18SRv: GTGCTGAAGGAGTCGTTTATAAAGA	620	Hodžić et al. 2015
Anaplasmataceae	16S rRNA	EHR16SD: GGTACCYACAGAAGAAGTCC EHR16SR: TAGCACTCATCGTTTACAGC	345	Brown et al. 2001
Hemotropic mycoplasmas	16S rRNA	HBT-F: TACGGCCCATATTCCTACG HBT-R: TGCTCCACCACTTGTTCA	600	Criado-Fornelio et al. 2003
Filarioid nematodes	COI	H14FilaCOIFw: GCCTATTTTGATTGGTGGTTTTGG H14FilaCOIRv: AGCAATAATCATAGTAGCAGCACTAA	724	Hodžić et al. 2015

## Results

Of the 103 dogs sampled (50 male and 53 female, 94 estimated to be 1 year or older and nine less than 1 year), 29 originated from a shelter in the area of Kefala, 28 are from a shelter located in the area of Nea Anatoli, 40 samples were collected from a shelter in the area of Agios Nikolaos, and six dogs originated from the town of Ierapetra (Fig. 1).

**Fig. 2:**
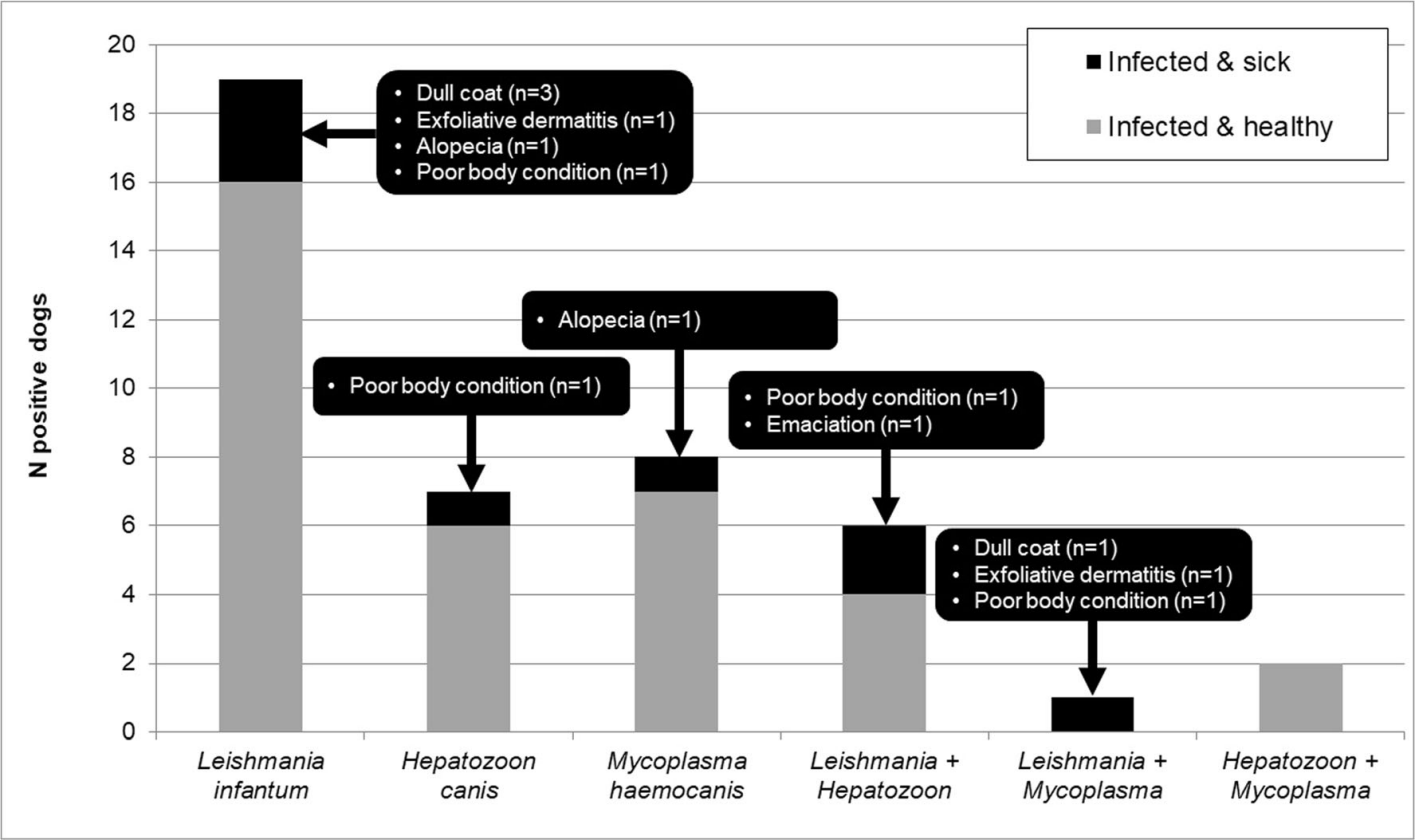
Number of dogs positive for one or two pathogens and their clinical condition

Overall, 43 dogs were positive for at least one pathogen, which corresponds to a prevalence of 41.7% (95% confidence interval [CI_95_] 32.7–51.4%) (Fig. 2). Mixed infections with two pathogens were observed in nine dogs (8.7%; CI_95_ 4.7–18.8%). In particular, sera from 26 dogs tested positive for *L. infantum* antibodies (25.2%; CI_95_ 17.9–34.4%) and five of these individuals displayed clinical signs which could be related to the infection (Fig. 2). However, no dog was positive in the PCR assays targeting *Leishmania* kinetoplast DNA (kDNA), small subunit ribosomal RNA (*ssu-rRNA*), and cysteine-proteinase B (*cpb*) genes.

*Hepatozoon canis* was detected in the blood of 15 dogs (14.6%; CI_95_ 9.0–22.7%) examined by PCR (*18S rRNA* gene). Sequence analyses revealed two different genetic variants; 14 sequences were identical to each other and displayed 100% homology to *H. canis* genotypes (GenBank®, e.g., KC138532) and one showed a 99% identity to, e.g., KX712126 genotype. One dog infected with *H. canis* had a poor body condition. Six dogs additionally had anti-*Leishmania* antibodies detected in the SNAP® test from which two individuals showed clinical disease manifestations (Fig. 2).

PCR assay targeting the *16S rRNA* gene of hemotropic mycoplasmas delivered positive results in 11 samples (10.7%; CI_95_ 6.1–18.1%) and *Mycoplasma haemocanis* (100% homology to *M. haemocanis* sequence KY117659) was confirmed by sequencing with complete homology for all obtained sequences. One of the *Mycoplasma*-infected dogs showed alopecia, and one dog co-infected with *Leishmania* showed alterations of the skin and coat and a reduced body condition (Fig. 2). Other pathogens tested for were not confirmed by using molecular and/or serological tools.

No ectoparasites were observed on the animals.

## Discussion

This study was performed to investigate the occurrence of important canine vector-borne pathogens on the island of Crete, where no data are available on these infections so far. Despite the small study population, three out of the six pathogens tested for could be detected, and the study revealed a high number of positive animals overall, and, to the best of our knowledge, reports the occurrence of *H. canis* and *M. haemocanis* for the first time on this Greek island.

In total, 25.2% of the dogs were positive for *L. infantum* antibodies, which is in accordance with the national mean seroprevalence previously reported in owned and stray dogs (Dujardin et al. 2008; Ntais et al. 2013). *Leishmania* DNA, however, was not detected by PCRs. This could be explained by the low sensitivity of PCR performed using peripheral blood as substrate, compared to spleen or lymph nodes aspirates (Reale et al. 1999). Moreover, dogs may also become PCR negative over time while specific antibodies persist for several months (Otranto et al. 2010). According to the data available from the late 1950s, phlebotomine sandflies, which are recognised vectors of *Leishmania* parasites, were nearly absent in Crete (Hadjinicolaou 1958), but they have re-emerged with growing population density reported during the last 30 years (Christodoulou et al. 2012). This resulted in an increasing number of human cases reported. In particular, 66 cases of human visceral leishmaniasis and 19 cases of cutaneous leishmaniasis had been recorded on this island between 1986 and 2010 (Christodoulou et al. 2012). According to a previous study, *L. infantum* was the predominant species found in human patients in Greece, while *L. tropica* could be confirmed only in the island of Crete (Ntais et al. 2013). Serology used in dogs does not differentiate between these two species (Baneth et al. 2017). The re-emergence of sandfly-borne infections in the Mediterranean is of some concern (Moriconi et al. 2017), and dogs play a pivotal role as reservoirs for zoonotic *Leishmania* species (Miró and López-Vélez 2018).

The prevalence rates of *H. canis* (14.6%) and *M. haemocanis* (10.7%) in the examined dogs from Crete were notably higher than those observed in studies from continental Greece (0.7% and 5.6%, respectively; Jensen et al. 2003; Tennant et al. 2011). Almost all of the dogs from this study were infected with the same *H. canis* genotype previously reported in cats (Baneth et al. 2013) and foxes (Criado-Fornelio et al. 2006). This finding indirectly suggests the occurrence of common transmission pathways between domestic and wild animals on the island. In contrast to many other CVBD-causing pathogens, hemotropic mycoplasmas are known to be transmitted not only by blood-sucking arthropods, but also directly via infected blood (Willi et al. 2010). Concomitant infections and immunosuppression presumably induce acute onset of disease (Baneth et al. 2001; do Nascimento et al. 2012) or exacerbation of clinical symptoms (Morgado et al. 2016). The zoonotic potential of other hemotropic mycoplasmas has been demonstrated in several human cases (Steer et al. 2011; Dos Santos et al. 2008; Yuan et al. 2009). According to a previous study, patients with extensive arthropod or animal contact, like veterinarians and veterinary technicians, are particularly at risk of infection (Maggi et al. 2013)*.*

Neither tick-borne bacteria from the family Anaplasmataceae, i.e., *Anaplasma phagocytophilum*, *Anaplasma platys*, *Candidatus Neoehrlichia mikurensis*, or *Ehrlichia canis*, nor *Babesia vogeli* was detected in the present study, which is quite surprising as the vector tick species *Ixodes ricinus* and *Rhipicephalus sanguineus*, responsible for the respective transmission, are known to occur on the island (Chochlakis et al. 2009; Dimanopoulou et al. 2017). This might be either due to the small sample size tested or to a low capacity of local *Rh. sanguineus* lineages to transmit *A. platys* or *E. canis* (Moraes-Filho et al. 2015; Diakou et al. 2018). Filarioid nematodes, including *Dirofilaria* spp., could also not be confirmed by PCR or (for *D. immitis*) antigen testing. However, among 200 dogs tested on four Greek islands, only one case of an imported *D. immitis* infection was reported, indicating that endemic infections by these parasites are most likely absent in insular Greece (Diakou et al. 2018).

Variations in the occurrence and prevalence of the tested pathogens compared to studies from continental parts of Greece may originate from the different climate conditions between northern and southern Greece and consequently differences in vector abundance. The fact that Crete is an island and the circulation of infected animals and vectors is limited may also explain the variable pathogen distribution and infection rates.

In the present study, 10 dogs showed clinical signs indicative of CVBD (alterations of the coat or skin or reduced/poor body condition), four of these were infected with one and three with two pathogens, and so overall clinical signs were observed in 16.3% of the 43 infected animals and 5.0% of the 60 uninfected dogs. Around 20% of the *Leishmania*- or *Hepatozoon*-positive dogs and three out of nine double-positive animals were clinically affected. Although the study population was too small to draw definitive conclusions, a trend for the development of clinical disease was seen in dogs infected with several (two) pathogens compared to one and the odds for uninfected dogs to show clinical signs were the lowest.

In conclusion, our study demonstrated that several blood-associated pathogens, including those of veterinary and public health importance, circulate among the dog population in Crete. Furthermore, we also reported the first occurrence of *H. canis* and *M. haemocanis* on this island. Results herein presented are of a great importance for the local veterinarians who seemed largely unaware of these canine pathogens. Our findings could possibly improve the way of routine examinations and prophylactic treatment in this region, especially in the light of a high rate of clinically unaffected infected dogs that may act as pathogen carriers. Along this line, dissemination of infectious agents (including CVBD) to non-endemic countries (and, in the worst case, long-term establishment) is considered a significant animal and public health threat promoted by travelling pets and the—often illegal—transfer of animals from the Mediterranean areas to Central Europe (Jensen et al. 2003; Menn et al. 2010; Fooks and Johnson 2015; Cito et al. 2016; Rijks et al. 2016). Precise knowledge on the distribution of CVBD significantly supports measures for prevention of their spread.
